# Artificial Intelligence in Orthodontics: Critical Review

**DOI:** 10.1177/00220345241235606

**Published:** 2024-04-29

**Authors:** N.F. Nordblom, M. Büttner, F. Schwendicke

**Affiliations:** 1 Department of Oral Diagnostics, Digital Health and Health Services Research, Charité–Universitätsmedizin Berlin, Berlin, Germany; 2 Department of Conservative Dentistry and Periodontology, University Hospital, Ludwig-Maximilians University of Munich, Munich, Germany

**Keywords:** deep learning/machine learning, orthodontic(s), big data, AI, computer vision/convolutional neural networks, treatment planning

## Abstract

With increasing digitalization in orthodontics, certain orthodontic manufacturing processes such as the fabrication of indirect bonding trays, aligner production, or wire bending can be automated. However, orthodontic treatment planning and evaluation remains a specialist’s task and responsibility. As the prediction of growth in orthodontic patients and response to orthodontic treatment is inherently complex and individual, orthodontists make use of features gathered from longitudinal, multimodal, and standardized orthodontic data sets. Currently, these data sets are used by the orthodontist to make informed, rule-based treatment decisions. In research, artificial intelligence (AI) has been successfully applied to assist orthodontists with the extraction of relevant data from such data sets. Here, AI has been applied for the analysis of clinical imagery, such as automated landmark detection in lateral cephalograms but also for evaluation of intraoral scans or photographic data. Furthermore, AI is applied to help orthodontists with decision support for treatment decisions such as the need for orthognathic surgery or for orthodontic tooth extractions. One major challenge in current AI research in orthodontics is the limited generalizability, as most studies use unicentric data with high risks of bias. Moreover, comparing AI across different studies and tasks is virtually impossible as both outcomes and outcome metrics vary widely, and underlying data sets are not standardized. Notably, only few AI applications in orthodontics have reached full clinical maturity and regulatory approval, and researchers in the field are tasked with tackling real-world evaluation and implementation of AI into the orthodontic workflow.

## Introduction

**Figure 1.**
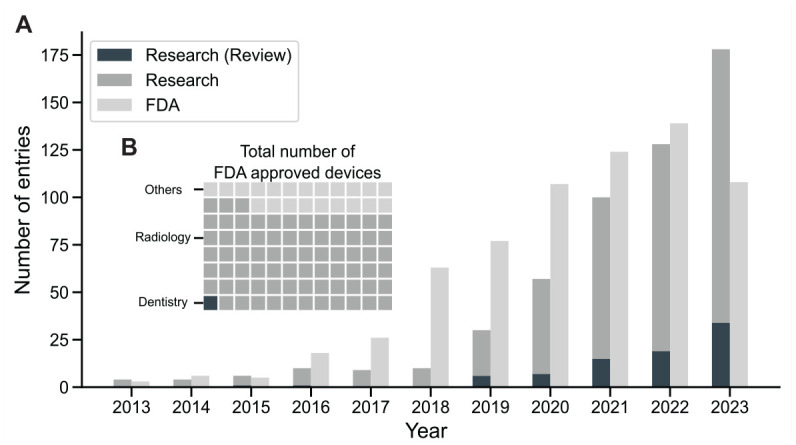
AI’s growth in medicine: Orthodontic research surges and broad FDA approvals. (**A**) The stacked bar plot shows a year-over-year increase in publications covering artificial intelligence in orthodontics. The data were obtained from a PubMed query covering the use of artificial intelligence in orthodontics and filtered by article type (Appendix Table 1). Dark gray bars represent review articles, and midgray bars represent nonreview articles in the field of orthodontics. Light gray bars indicate the annual count of machine learning–enabled medical devices approved by the Food and Drug Administration (FDA) across all medical fields. (**B**) Of the total number of medical devices approved by the FDA as of July 2023, most are in the field of radiology and only a small subset is attributed to dentistry. Each square corresponds to 7 approved devices.

While the interest in advanced data-analytic methods, such as artificial intelligence (AI) and machine learning, is rising in the field of orthodontics, the number of regulatorily approved applications employing AI trails the number of publications (Fig. 1A). As of July 2023, the US Food and Drug Administration (FDA) had approved 676 AI and machine learning–enabled medical devices, of which just over 1% were related to dentistry: 6 in dental radiology and 1 in orthodontics (CEREC Ortho Software; Dentsply Sirona) (Food and Drug Administration 2023) (Fig. 1B). However, a multitude of scoping reviews are painting a positive outlook for the use of AI in orthodontics. The use cases presented therein are multifaceted and promising: AI could help orthodontists with the evaluation of clinical imagery (e.g., landmark detection in lateral cephalograms), providing decision support (need for orthodontic extraction, need for orthognathic surgery, outcome prediction, and others) and partial relief from routine tasks (documentation, remote follow-ups) (Liu et al. 2023).

This first part of this Critical Review is aimed at appraising hot topics in the field of AI in orthodontics. Further, recent technical opportunities such as multimodal learning or large language models (LLMs) are presented, and their impact on orthodontics is discussed. The second part of this review focuses on limitations, risks, and challenges that need to be overcome to guarantee a safe use of AI in orthodontic practice. Guidance is provided on how researchers can contribute to improving the quality, replicability, and reporting of studies on AI in orthodontics.

## Applications

Orthodontic data sets pose a great potential for the application of AI because of their standardized, longitudinal, and multimodal data set structure. A range of AI applications are available in orthodontics. The following section critically evaluates the status of these applications and gives directions on further development.

### Analysis of Lateral Cephalograms

One of the most researched tasks in orthodontics is automated lateral cephalogram landmark detection. Conventionally, using these landmarks, specific parameters are deduced and employed for orthodontic treatment planning and evaluation. Automated AI-based landmarking has made significant advancements: in the “2015 IEEE Grand Challenge in Evaluation and Comparison of Anatomical Landmark Detection Methods for Cephalometric X-ray Images,” the reported successful detection rate of landmarks (detection within a 2-mm precision range from the reference test landmarks) was at 70% (Wang et al. 2015). In 2022, a study evaluating a commercial product (CephX; ORCA Dental AI) reported a successful detection rate of 98% (Davidovitch et al. 2022).

**Figure 2.**
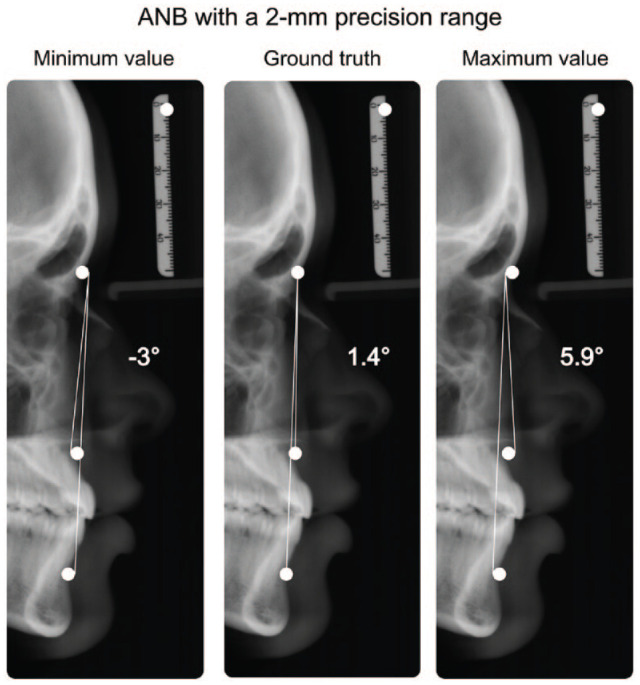
Exemplary depiction of different metrics used to describe artificial intelligence landmark detection: all landmarks (A point, nasion, B point) are located within the 2-mm detection range (white circle) of the ground truth, while the clinically relevant parameter ANB exhibits a range of 8.9°.

While the 2-mm precision range is considered clinically acceptable, the cumulative error when calculating clinically relevant parameters can be substantial. For example, the ANB angle—measuring the anteroposterior relationship between the maxilla and mandible—tolerates large errors for the detection of the A and B points in the vertical but not the horizontal plane (Schwendicke, Chaurasia, et al. 2021). It may vary by over 8° (i.e., double the 2.0 ± 2.0° range indicating a normal sagittal relationship between the maxilla and mandible), even though all 3 defining landmarks (A point, nasion, B point) are detected within a 2-mm precision range from the ground truth, as depicted in Figure 2.

A recent study evaluated the quality of commercially available products and reported significant differences compared to the human gold standard for the clinically relevant parameters (Kunz et al. 2023; Kunz, Stellzig-Eisenhauer, Widmaier, et al. 2023). Among others, CephX, the product mentioned above (with a 98% successful detection rate), showed significant deviations from the human gold standard in 5 of 9 investigated parameters.

Moreover, even for this identical task—cephalometric landmark detection—studies in the field use highly variable AI methods and report their findings using a range of metrics, scales, and aggregations, as indicated by a recent scoping review that included 39 publications (Liu et al. 2023). The result of this heterogeneity is limited comparability of studies. Researchers are encouraged to report model performances (e.g., successful prediction within a specific threshold, mean radial error) along with the performances of clinically used parameters and diagnoses in standardized formats (Norgeot et al. 2020).

As the eventual aim of landmark detection is to derive clinically useful diagnoses (e.g., classification of growth types) that help guide orthodontists toward treatment decisions, end-to-end AI applications have been developed to directly classify lateral cephalograms based on clinically relevant diagnoses. Here, the neural networks learn relevant features from labeled images indicating a specific class without the need for landmark tracing. AI-based classification of vertical and sagittal skeletal patterns has been found highly accurate, with a mean area under the receiver operating characteristic curve (ROC AUC) of >95% (Yu et al. 2020).

In a similar fashion, lateral cephalograms were used for end-to-end predictions of the upper airway obstruction (Jeong et al. 2023), degenerative temporomandibular joint diseases (Fang et al. 2023), or the classification of cervical vertebrae maturation stages (Atici et al. 2023). Notably, such end-to-end classifications are not inherently explainable, that is, humans cannot easily identify why a certain class was chosen, something we critically discuss below.

Most of the published research within the field of automated landmark detection made use of unicentric data for model training and evaluation. This can lead to overfitting, where the model learns both the underlying pattern and the random noise (e.g., reduced operator variability, image processing artifacts, or machine calibration specifics) in the training data set (Mutasa et al. 2020). Training the model on such a homogeneous data set can lead to poor predictive performance on new data. Thus, further validation on diverse, multicentric data sets remains necessary, a point that we expand below.

### Assessment of Orthodontic Treatment Need and Detection of Contraindications

The detection of orthodontic treatment need is based on standardized indices that weigh and summarize a variety of diagnostic findings and usually relies heavily on the analysis of the tooth position. Out of 31 categories described by the Index of Orthodontic Treatment Need (IOTN) (Brook and Shaw 1989), 24 require the analysis of tooth position. Thus, the analysis of dental models represents a task of high relevance.

Automatic segmentation of teeth in digital dental models has significantly improved over the past years (Ben-Hamadou et al. 2023) and serves as a backbone for further automated measurements and analysis. To compare different AI-based tooth segmentation and tooth labeling models, a first multicentric benchmarking data set has recently been published following the “3DTeethSeg’22 challenge” (Ben-Hamadou et al. 2023), involving 1,800 labeled intraoral scans. Scanning was performed using 3 different commercially available intraoral scanners to increase generalizability. The best-performing model yielded an excellent segmentation accuracy of 0.99 (Ben-Hamadou et al. 2023).

Despite these advances in automatic assessment of teeth and their position, discerning the need for orthodontic treatment remains challenging, particularly in borderline cases. In such instances, the interexaminer variability is often substantial for remote case assessment (κ values between 0.16 and 0.37) but also for on-site clinical assessments (0.22–0.38) (Baelum et al. 2012). Such variability can present significant hurdles for model training and performance, consequently complicating the integration of AI into the process (Kang et al. 2022). A statistically significant relationship for the detection of orthodontic treatment need between the remote assessment and the on-site clinical examination was found even if the remote assessment was conducted solely using clinical photographs and dental casts (Baelum et al. 2012). Overall, it is well conceivable that AI could help clinicians identify orthodontic treatment need.

Moreover, AI can help not only to diagnose orthodontic findings but also to detect contraindications for orthodontic treatment, like the presence of active carious lesions, apical lesions, or periodontal bone loss. Markedly, the detection of these pathologies is widely studied in the field of AI in dentistry (Arsiwala-Scheppach et al. 2023), and a range of software applications providing such detection functionality has entered the market recently. These can assist orthodontic specialists during treatment planning while supporting automated documentation and patient communication (Kosan et al. 2022).

### Patient Compliance and Communication

AI and Big Data offer promising solutions to enhance patient engagement, specifically addressing the often-challenging “last mile” problem in health care (Davenport and Kalakota 2019). Originating from the logistics industry, in medicine the term *last mile* refers to the last step needed for successful care delivery. It is affected by aspects such as accessibility, patient expectation, and information, as well as communication and compliance. Here, therapeutic success with removable orthodontic appliances greatly depends on wearing time (Torsello et al. 2022). The potential of following patients up remotely and thus increasing therapy adherence has been proven in other fields of medicine (Babel et al. 2021).

AI holds the potential to revolutionize the way patient follow-ups are conducted, using remotely obtained data from wearable devices or image sources (Prasad et al. 2023). The application of AI in telemonitoring software has been positively received by both patients and dental practitioners alike (Dalessandri et al. 2021). First applications leveraging AI for remote monitoring in clear aligner therapy have been introduced. Yet, these have faced limitations due to inconsistencies in the instructions provided to the patients (Ferlito et al. 2023). Furthermore, ethical and legal questions arise when AI-driven apps alone provide medical instructions to the patient, omitting medical experts in the process (Schneeberger et al. 2020).

AI-based speech analysis using natural language processing (NLP) could support patient communication (Büttner et al. 2024). NLP targets the intersection between informatics and linguistics by making computers process and “understand” human language. A popular application is the use as an AI chatbot. With recent advancements in the development and availability of models like generative pretrained transformers (GPTs), such as GPT-4 (OpenAI) or Google Bard (Google), persuasive conversations with AI are possible. The unique features of these so-called foundation models are that multiple (currently nonmedical) downstream tasks such as coding, translating, and sentiment analysis are possible with a single network architecture. In orthodontics, such models have the potential to be applied by clinicians to assist with treatment decision support, support with administrative tasks, or interactive notetaking. Notably, large language models (LLMs) like GPT-4 are not designed to serve as medical devices (yet) due to their shortcomings in areas like explainability and transparency, as well as the lack of comprehensive oversight and validation systems (Moor et al. 2023). It has been demonstrated that ChatGPT, along with other commercially available LLMs, has an unpredictable ability to convincingly produce invented content, a phenomenon known as hallucination (Gilbert et al. 2023). Here, knowledge injection can be applied to reduce the number of hallucinated responses while infusing models with context-specific information (Martino et al. 2023).

Similar to generating text, generative AI can help with patient communication through image generation. First efforts have been undertaken to predict changes in an adult’s facial morphology following orthodontic treatment using AI (Park et al. 2022). Intermediate fusion, a multimodal fusion technique that we describe below, was used to combine data from pretreatment cone beam computed tomography (CBCT) together with patient characteristics (i.e., patients’ age, sex, and the amount of incisor movement provided by orthodontic treatment). Most notably, more than half of the surveyed experienced orthodontist were unable to distinguish between the artificially generated and the real facial profile.

### Multimodal Learning in Orthodontic Treatment Planning

**Figure 3.**
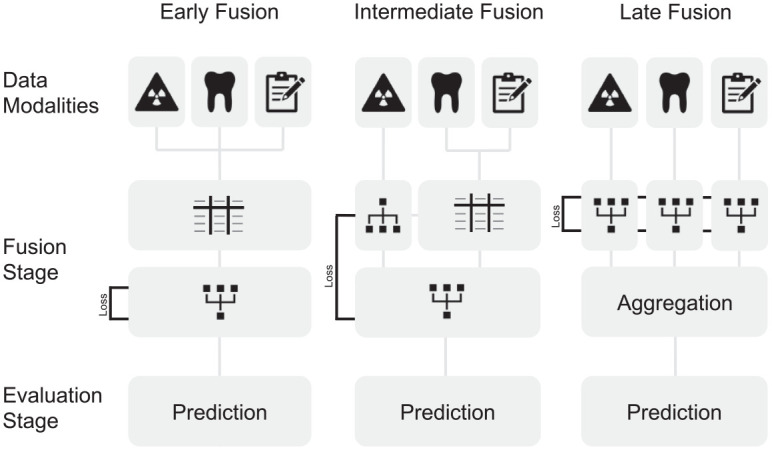
Illustration of data fusion techniques that can be applied for orthodontic data sets. Early fusion requires handcrafted features in a common data format (represented as a table) for learning. Subsequentially, a network that allows for intermodal abstractions is trained on these data. In intermediate fusion, loss, a main component to the network learning ability, is propagated back to the feature extraction layer, potentially allowing for features extracted from the data that surpass human experts. In late fusion, loss is computed individually for each model extracting features from the data. The output of the individual networks is then aggregated.

Orthodontists make use of multiple data sources for orthodontic treatment planning. Similarly, multimodal learning can be used in the field of AI. Here, different types of data such as radiographic images, text, and numerical metrics can be combined into one model to predict an outcome, as illustrated in Figure 3.

The stage when data are combined and fed into the model is called the fusion stage. Early fusion marks the most straightforward approach to the fusion techniques: data of different modalities are combined through translation into a common data format. For example, features extracted from lateral cephalograms, clinical data, and clinical notes are concatenated in a single tabular data format prior to model training (Fig. 3). While simple and efficient, this fusion technique relies on handpicked features from each modality that implicate expert knowledge and thus incorporate a bias. Intermediate fusion reduces this bias by combining modality-specific features while incorporating a neural network, extracting features from a single or multiple input modalities. The features extracted from the neural network can thus change with every iteration of training, leading to better model performance. In late fusion, distinct models are trained to extract features from different modalities. While powerful, this method makes the final decision based on an aggregation function of all models and is potentially missing cooperative information that could be abstracted from the combination of different data modalities (Huang et al. 2020; Acosta et al. 2022).

First applications of multimodal AI in orthodontics assessed the necessity of orthognathic surgery (Choi et al. 2019) or the necessity of tooth extractions (Suhail et al. 2020; Real et al. 2022) using early fusion models. In the latter study, the data set for model training was constructed with features obtained from manual landmark detection in lateral cephalograms and numeric features extracted from dental casts to predict the necessity of tooth extractions. The AUC improved when the model was trained using both modalities compared to just 1 modality (Real et al. 2022).

Once again, validation of the proposed models remains crucial and is currently lacking. Data sets are constructed using unicentric data; in some studies, only 1 expert provided the treatment decisions (labels) (Choi et al. 2019; Real et al. 2022). As interrater variability is assumed to be substantial for both the assessment of tooth extraction need (Suhail et al. 2020) and orthognathic surgery need (Kunz, Stellzig-Eisenhauer, and Boldt 2023), more extensive data sets are necessary that allow for generalizability of the proposed algorithms.

Notably, efforts toward intermediate or late fusion have only been undertaken for tasks such as classification of lateral cephalograms or treatment outcome prediction (Yu et al. 2020; Park et al. 2022). However, the application of intermediate or late fusion techniques in orthodontic treatment planning has not been assessed, but it may be worthwhile, as features extracted from distinct modalities could be more meaningful than those captured by human experts (Acosta et al. 2022).

### Precision Orthodontics

Precision medicine makes it possible to deliver customized care to patients, such as individualized drug dosage to increase treatment efficiency and reduce harmful side effects. In oncology, precision medicine already is in clinical use: for example, next-generation sequencing allows to determine specific mutations in melanoma along key pathways, and treatment is subsequentially individualized by tailoring drug application for patients with specific mutations (Flaherty et al. 2012). Similarly, orthodontic patients could benefit from treatment plans that are individually tailored to their unique circumstances. Genetic polymorphisms in the catechol-O-methyltransferase (COMT), for example, have been shown to be associated with temporomandibular disorder, and thus more conservative treatment options could be considered in patients with these polymorphisms (Brancher et al. 2021). Furthermore, speed of orthodontic tooth movement based on individual bone densities (Chugh et al. 2013) or type and length of the retention phase could be individualized.

While growth prediction and response to treatment can be highly individual, AI could leverage more sophisticated algorithms to predict growth and to derive, for example, the optimal time point for treating class II patients with removable functional appliances (Perinetti et al. 2015).

Precision medicine is data hungry, so conventional orthodontic data sets could be expanded by genetic, epigenetic, proteomic, metabolic, and other “omic-based” data (Schwendicke and Krois 2022; Schwendicke F, Krois J. 2022b). Radiomics, extracting features from radiographs that are not visible to the human eye, can play a role in this development—the detection of ankylotic teeth in radiographs being one relevant example.

## Challenges Ahead

The outlook for AI in orthodontics looks promising and manifold, with applications such as accurate and streamlined diagnostics, decision support systems, patient communication, and precision orthodontics, as presented above. Nonetheless, there are still significant obstacles that need to be overcome for its wider implementation. The subsequent section explores these challenges and proposes initial solutions to address them.

### Generalizability and Data Quality

As laid out above, most AI algorithms in orthodontics are currently trained and evaluated on unicentric data, encompassing the inherent risk of bias (Krois et al. 2021). Particularly in orthodontics, studies have demonstrated that parameters in lateral cephalograms can exhibit substantial variation among patients from diverse populations (Ajayi 2005). Further efforts should be dedicated to the development of well-balanced data sets to reduce sample selection bias and ensure equitable and generalizable algorithms, while increasing heterogeneity and the representation of the manifold. Generalizability can be increased by expanding data set size and heterogeneity. However, this is difficult to accomplish for medical data sets for several reasons, with data privacy and cost of annotations, among others. Here, it is crucial to add that data quality of both the raw data and the annotations plays a crucial role for AI performance (Gunasekar et al. 2023). Approaches like federated learning, a method we discuss below, may assist in overcoming some of these issues, particularly those related to data privacy and access.

### Data Privacy, Transparency, and Permission

Orthodontic data sets represent a considerable privacy and security risk due to the sensitive nature of the data they contain—among others, clinical photographs that can make patients identifiable. Furthermore, different data modalities are usually stored in isolated repositories, referred to as “data silos” (Rischke et al. 2022); accessing them and fusing them remains challenging.

Balancing the need to access the wealth of data, also across silos, to achieve sufficiently broad and deep (i.e., generalizable) data sets while maintaining data privacy may be addressed by federated learning. The method permits data to remain at their original site while optimizing a centralized algorithm by federating what was learned from each decentral data set. This approach can be used to comply with local privacy constraints while increasing model generalizability. In radiology, federated learning has been successfully employed for segmenting glioblastomas in magnetic resonance imaging, with significant improvements in model performance and generalizability across centers (Pati et al. 2022). First approaches in dentistry to employ federated learning have been made, too (Schneider et al. 2023). To increase data diversity, orthodontics could profit from federated learning alike, especially in fields where case numbers in single research centers are low (such as for most craniofacial malformations and other rare conditions).

### Explainability and Accountability

Explainability refers to the black box phenomenon of AI. Deep learning algorithms inherently lack the ability to explain model predictions. Thus, efforts have been undertaken that provide insight into this “black box.” AI-based end-to-end classification of lateral cephalograms (e.g., growth pattern, cervical vertebrae maturation stages) is not inherently explainable. Here, methods such as gradient-weighted class activation mapping (Grad-CAM) (Selvaraju et al. 2020) are widely used in research, allowing insights into the features that the AI considers important for its decision-making. Alignment with features experts consider important can increase trust.

Currently, a discussion is being conducted in the field of medicine about whether medical AI must be explainable. Some critics claim that AI must not be inherently explainable, comparing the application of AI in medicine to drugs, where the concrete mechanism of action is not (yet) understood. However, unexplainable AI systems can be prone to several biases, with selection bias being one of them (Schwendicke and Krois 2022; Schwendicke F, Krois J. 2022a). As the dental specialist is responsible for the final treatment choice, practitioners must be fully aware of these and other fundamental concepts of AI when applying such algorithms in practice (Schwendicke et al. 2023).

### Interoperability

Interoperability lays the foundation to a frictionless integration of AI into the clinical workflow. It simplifies the development of AI in orthodontics and streamlines the application in the clinical workflow (Lehne et al. 2019). Interoperability becomes more crucial for the development and deployment of multimodal AI. Currently, however, the majority of clinical notetaking in orthodontics lacks standardization. This poses a significant challenge in consolidating these valuable data into data sets for model training. Interoperability could be increased by standardizing data storage formats and implementing systems optimized for interoperability, such as the Systematized Nomenclature of Dentistry (SNODENT), Digital Imaging and Communications in Medicine (DICOM), or Fast Healthcare Interoperability Resources (FHIR). The use of interoperable data formats, such as FHIR, would facilitate the validation and application of developed algorithms in real-world scenarios (Lehne et al. 2019). Additionally, the effectiveness of techniques like federated learning could be significantly enhanced through the standardization of data formats. However, given that all these formats mentioned are internationally defined, they often require locally translated terminologies. Further, a solution to safely combine data protection and interoperability is currently missing. Thus, the implementation of such nomenclature and harmonized formats is limited.

### AI in Orthodontic Research

The widely adopted “FAIR Guiding Principles for Scientific Data Management and Stewardship” (Findability, Accessibility, Interoperability, and Reusability) outline good practice in the management of digital assets (Wilkinson et al. 2016). In short, data (and metadata) in research should be made machine findable by providing rich metadata to data sets; accessible with appropriate authentication and authorization methods; interoperable, allowing for smooth integration with other data; and reusable, meaning that data are well described with domain-relevant community standards. These principles are widely adopted and encouraged by journals (Celi et al. 2019).

In machine learning research, open code and open data are important to guarantee reproducibility and validation. GitHub and GitLab are currently among the most popular platforms used to distribute code accompanying papers. Open data, on the other hand, pose a major challenge when identifiable patient data are made publicly available. To overcome this issue, deidentified, synthetic data sets have been discussed as a possible solution. In orthodontics, generative adversarial networks (GANs) have been applied to generate synthetic frontal images of patients (Tian et al. 2023). While promising, medical imaging data sets created by GANs currently are not sufficient for training purposes (Skandarani et al. 2023).

Presently, the whole dental field, including orthodontics, is lacking curated “benchmarking” data sets (allowing to test AI software on standardized, representative data sets and hence to compare different applications in a useful way) and uniform reporting metrics (Arsiwala-Scheppach et al. 2023). Both aspects complicate the task of comparing and assessing orthodontic AI. Although data sets for specific tasks like tooth segmentation in dental models, landmark detection in lateral cephalograms (as presented above), and panoramic radiographs (Zhang et al. 2023) are accessible, curated, comprehensive, multimodal data sets on par with resources like the MIMIC-III data set used in critical care are missing. The availability of such resources would significantly contribute to the advancement and standardization of AI in orthodontic research.

On the other hand, sharing models and model weights alongside publications offers a viable, more privacy-oriented alternative to open data. Sharing trained models allows reviewers and researchers to test AI models on local data sets. Platforms such as PapersWithCode, Kaggle, or HuggingFace are dedicated to facilitating the distribution of both code and AI models linked to scientific publications. However, the process of model validation remains labor-intensive and requires a significant amount of technical expertise often subject to local hardware constraints. This challenge becomes increasingly complex with multimodal AI, where models integrate diverse data types. Here again, interoperability is crucial.

Reproducibility and replication of machine learning models across study populations and research groups poses a major challenge, especially in the field of orthodontics. Here, sensitive patient data, which most of the time are inaccessible for replication by others, lay the foundation of machine learning models. Thus, comprehensive, systematic, and transparent reporting becomes crucial. Similar to CONSORT, which provides evidence-based recommendations for reporting of randomized controlled trials (Moher et al. 2010), adopting specific reporting guidelines tailored to the application of AI in medicine such as TRIPOD, MI-CLAIM, or CONSORT-AI can greatly enhance the quality of reporting in the field. For dentistry, a reporting guideline for AI studies with a focus on image analytics has been published, too (Schwendicke and Krois 2021). Reviewers, editors, and journals are encouraged to enforce application of such guidelines (Schwendicke and Krois 2021).

Moreover, readers, reviewers, and editors should scrutinize papers for selective reporting and “spin” (i.e., overinterpretation of positive research findings and underrepresentation of those findings that are less positive). Spin has been found in studies developing and testing machine learning models, too, for example, in oncology (Dhiman et al. 2023). Preregistration of studies and considerate application of AI and machine learning methods, including a critical stance toward the employed data sets, their representativeness, generalizability, breadth and fairness, and comprehensive and objective reporting of any findings, should be the standard in AI research in orthodontics.

## Conclusions

Given the large amount of standardized, longitudinal multimodal data available, application of AI in orthodontics is promising and multifaceted. Notably, the number of useable clinical applications trails that of publications. By increasing interoperability and data availability, as well as reducing hurdles for a seamless integration of AI into the clinical workflow, clinicians and patients could profit alike from its use. Here, multimodal learning, among other methods, could be used to increase diagnostic accuracy while advancing precision orthodontics. However, sophisticated mechanisms are needed to ensure data safety and data privacy.

When training AI algorithms in orthodontics, one should focus on constructing data sets with a sufficient heterogeneity to allow for generalizability and a reduction of bias. As explainability with deep learning methods such as foundation models becomes increasingly complex, rigorous evaluation and testing of such algorithms is more necessary.

Adherence to reporting guidelines is encouraged when publishing in the field of AI in orthodontics to increase transparency for both reviewers and readers. Moreover, there is a need for prospective clinical trials that evaluate the usability and performance of such algorithms in a clinical context.

## Author Contributions

N.F. Nordblom, contributed to conception, design, data acquisition, analysis, and interpretation, drafted and critically revised the manuscript; M. Büttner, F. Schwendicke, contributed to conception, design, data analysis and interpretation, critically revised the manuscript. All authors gave final approval and agree to be accountable for all aspects of the work.

## Supplemental Material

sj-docx-1-jdr-10.1177_00220345241235606 – Supplemental material for Artificial Intelligence in Orthodontics: Critical Review Supplemental material, sj-docx-1-jdr-10.1177_00220345241235606 for Artificial Intelligence in Orthodontics: Critical Review by N.F. Nordblom, M. Büttner and F. Schwendicke in Journal of Dental Research
