# Supplementary material for: Artificial Intelligence in Orthodontics: Critical Review
Source: J Dent Res. 2024 Apr 29;103(6):577–84. doi: 10.1177/00220345241235606 (PMC11118788; doi:10.1177/00220345241235606)
Supplement: sj-docx-1-jdr-10.1177_00220345241235606 – Supplemental material for Artificial Intelligence in Orthodontics: Critical Review [file sj-docx-1-jdr-10.1177_00220345241235606.docx]

Appendix

**Table 1** Annual count of research articles (including review and non-review articles) and review articles alone (research articles filtered for reviews and systematic reviews) listed on PubMed for the search query: ((“(orthodontics) AND ((machine learning) OR (artificial intelligence))”, accessed December 31, 2023. Number of AI and machine learning-enabled medical devices approved by the FDA in medicine and dentistry (Food and Drug Administration 2023). FDA listings have been manually screened; approvals categorized under the category radiology and covering dental diagnostics have been recategorized under dentistry.

| **Year** | **Research** |  | **Research (Review)** | **FDA approvals**  **in medicine** | **FDA approvals in dentistry** |
| --- | --- | --- | --- | --- | --- |
| 2023 | 178 |  | 34 | 108 | 2 |
| 2022 | 128 |  | 19 | 139 | 3 |
| 2021 | 100 |  | 15 | 124 | 1 |
| 2020 | 57 |  | 7 | 107 | 0 |
| 2019 | 30 |  | 6 | 77 | 0 |
| 2018 | 10 |  | 0 | 63 | 1 |
| 2017 | 9 |  | 0 | 26 | 0 |
| 2016 | 10 |  | 1 | 18 | 0 |
| 2015 | 6 |  | 1 | 5 | 0 |
| 2014 | 4 |  | 0 | 6 | 0 |
| 2013 | 4 |  | 0 | 3 | 0 |
